# Description of male *Tylorida sataraensis* Kulkarni, 2014 (Araneae, Tetragnathidae) with notes on habits and conservation status

**DOI:** 10.3897/BDJ.3.e4451

**Published:** 2015-02-16

**Authors:** Siddharth Shrikant Kulkarni, Todd R Lewis

**Affiliations:** † Biome Conservation Foundation, Pune, India; ‡ Zoology Department, Yashavantrao Chavan Institute of Science, Satara, Satara, India; § Westfield, 4 Worgret Road, Wareham, Dorset, BH20 4PJ., Dorset, United Kingdom

**Keywords:** Tetragnathid spider, laterite rocks, streams, seasonal surveys, population marginalisation, endangered species

## Abstract

The male sex of *Tylorida sataraensis* Kulkarni, 2014 is described based on specimens from the type locality. The distinguishing characters from its closest species *Tylorida ventralis* (Thorell, 1877) are detailed. An interesting behaviour of going underwater by *T. sataraensis*, on disturbance is recorded and tested for significance. The surveys have shown sighting of this species only to the perennial streams of the rocky outcrops in Satara region. The potential threats to this species and the possible conservation status based on known distribution are discussed.

## Introduction

The genus *Tylorida* Simon, 1894 was established on the basis of a male *Tylorida striata* (Thorell, 1877) whose holotype depository details are unknown (Álvarez-Padilla and Hormiga 2011). There are ten currently described species for *Tylorida* (World Spider Catalog 2014). Most of these are distributed in Australasia except for *Tylorida seriata* Thorell, 1899 that extends to West Africa and Cameroon (Álvarez-Padilla and Hormiga 2011). To date, three species of *Tylorida* are reported from India; *Tylorida culta* (O. P.-Cambridge, 1869), *Tylorida sataraensis* Kulkarni, 2014 and *Tylorida ventralis* (Thorell, 1877). *Tylorida culta* is also reported from Sri Lanka and *T. ventralis* has a wider distribution from India, to Taiwan, Japan and New Guinea.

## Materials and methods

Seven streamside transect surveys of up to 400m were conducted for spiders during 2012, 2013 and 2014. Specimens were collected by visual searching from perennial streams among secondary montane forest along *T. sataraensis* type locality; Chalkewadi (17.478°N, 73.836°E; 1078m ASL) and Kaas (17.721°N, 73.808°E; 1123m ASL) plateaus of Satara district, India. Males of the species were confirmed by observing copulation. Specimens were collected on private land whilst engaged on permitted surveys for other fauna. Specimens were examined using a Brunel IMXZ™stereozoom microscope and imaged using Canon 1200D™ mounted camera. Statistics were performed in Statsoft Statistica Ver. 7.0™ (StatSoft 2011​). Mapping was prepared using DIVA GIS™ v7.5c. All specimens were deposited at Bombay Natural History Society, Mumbai. All the morphological measurements are in millimeters. Abbreviations used: AME - anterior median eyes, PME - posterior median eyes, CDBP - Cymbial dorsobasal process, ASL - Above sea level, BNHS - Bombay Natural History Society, IUCN - International Union for Conservation of Nature.

## Taxon treatments

### Tylorida sataraensis

Kulkarni, 2014

urn:lsid:zoobank.org:pub:7CDB5A97-C7DB-452F-9BB8-0C5A9F071E9F

#### Materials

- **Type status:** Other material. **Occurrence:** catalogNumber: BNHS Sp. 119; recordedBy: V. Deshpande; individualCount: 1; sex: 1 male; lifeStage: Adults; **Taxon:** scientificName: Tylorida sataraensis; kingdom: Animalia; phylum: Arthropoda; class: Arachnida; order: Araneae; family: Tetragnathidae; genus: Tylorida; specificEpithet: sataraensis; taxonRank: species; scientificNameAuthorship: Kulkarni, 2014; taxonomicStatus: accepted; **Location:** continent: Asia; country: India; countryCode: IN; stateProvince: Maharashtra; municipality: Satara; locality: Chalkewadi; verbatimLocality: Chalkewadi sada; verbatimElevation: 1078 m; georeferenceVerificationStatus: Verified by collector; **Identification:** identifiedBy: Siddharth Kulkarni; dateIdentified: 08/05/2014; **Event:** samplingProtocol: Hand picking; eventDate: 05/06/2013; habitat: Rocky plateaus; **Record Level:** language: en; rightsHolder: Siddharth Kulkarni; institutionID: Bombay Natural History Society (BNHS), Mumbai; institutionCode: BNHS; collectionCode: Sp
- **Type status:** Other material. **Occurrence:** catalogNumber: BNHS Sp. 120-121; recordedBy: S. Kulkarni and A. Vartak; individualCount: 2; sex: 2 males; lifeStage: Adults; **Taxon:** scientificName: Tylorida sataraensis; kingdom: Animalia; phylum: Arthropoda; class: Arachnida; order: Araneae; family: Tetragnathidae; genus: Tylorida; specificEpithet: sataraensis; taxonRank: species; scientificNameAuthorship: Kulkarni, 2014; taxonomicStatus: accepted; **Location:** continent: Asia; country: India; countryCode: IN; stateProvince: Maharashtra; municipality: Satara; locality: Chalkewadi; verbatimLocality: Chalkewadi sada; verbatimElevation: 1078 m; georeferenceVerificationStatus: Verified by collector; **Identification:** identifiedBy: Siddharth Kulkarni; dateIdentified: 08/05/2014; **Event:** samplingProtocol: Hand picking; eventDate: 04/19/2014; habitat: Rocky plateaus; **Record Level:** language: en; rightsHolder: Siddharth Kulkarni; institutionID: Bombay Natural History Society (BNHS), Mumbai; institutionCode: BNHS; collectionCode: Sp
- **Type status:** Other material. **Occurrence:** catalogNumber: BNHS Sp. 122 & 123﻿; recordedBy: S. Kulkarni; individualCount: 2; sex: 2 males; lifeStage: Adults; **Taxon:** scientificName: Tylorida sataraensis; kingdom: Animalia; phylum: Arthropoda; class: Arachnida; order: Araneae; family: Tetragnathidae; genus: Tylorida; specificEpithet: sataraensis; taxonRank: species; scientificNameAuthorship: Kulkarni, 2014; taxonomicStatus: accepted; **Location:** continent: Asia; country: India; countryCode: IN; stateProvince: Maharashtra; municipality: Satara; locality: Kaas; verbatimLocality: Kaas sada; verbatimElevation: 1123 m; georeferenceVerificationStatus: Verified by collector; **Identification:** identifiedBy: Siddharth Kulkarni; dateIdentified: 08/05/2014; **Event:** samplingProtocol: Hand picking; eventDate: 02/14/2014; habitat: Rocky plateaus; **Record Level:** language: en; rightsHolder: Siddharth Kulkarni; institutionID: Bombay Natural History Society (BNHS), Mumbai; institutionCode: BNHS; collectionCode: Sp

#### Description

Total length: 8.1-9.7; carapace: 3.91-4.27 long, 2.31-2.48 wide; abdomen: 3.89-4.01 long, 1.78-1.89 wide. Body pattern in male similar to its female (Kulkarni 2014) (Figs 6, 7). *Cephalothorax*. Cephalic and thoracic region in same plane. Lateral eyes encircled black, placed on prominent tubercles. AME separated by its diameter, PME less than its diameter. Smooth black pubescence on carapace. Thoracic region margined dark black. Chelicerae brown, long with three promarginal and four retromarginal teeth. Labium brown, semi-circular with slight corrugations; endites longer than wide, with wider proximal edge. Sternum brown, overall heart-shaped with straight margin at coxa II and folded inwards at coxae III and IV. Legs yellow coloured, femora black distally. *Abdomen* oval shaped, narrower than cephalothorax and slightly overlapping thoracic region when viewed laterally. Dorsum covered with greenish pubescence; venter black with thick yellow lines on the margin, sparsely covered with silver specks and whitish pubescence.

Cymbial dorso-basal process is shorter than half the cymbial width and perpendicular to cymbium longitudinal axis. Embolar base roughly circular. Long macrosetae on palpal metatarsus measuring half times its length. Morphometry of palpal organs is given in Table 1.

##### Remarks

The variation in body size and patterns in *T. ventralis* (see Jäger and Praxaysombath 2009) may be confused with *T. sataraensis*. Male palp and female epigyne examination can confirm the species identity. Furthermore, we found webs of *T. sataraensis* exclusively above and across stream water surfaces whereas webs of *T. ventralis* were found either adjacent to stream margins or away, among low vegetation; both observed in different localities.

#### Diagnosis

**Figure 1.**
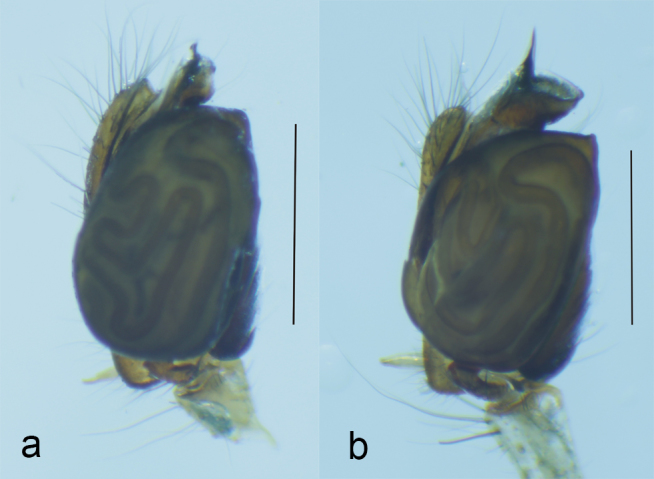
Right palp, dorsal view of (a) *T. ventralis* and (b) *T. sataraensis* (Scale = 1mm).

**Figure 2.**
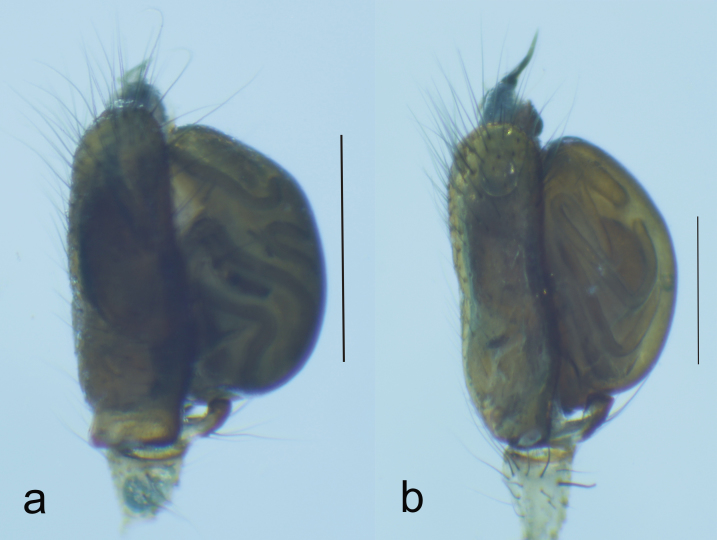
Right palp, ventral view of (a) *T. ventralis* and (b) *T. sataraensis* (Scale = 1mm).

**Figure 3.**
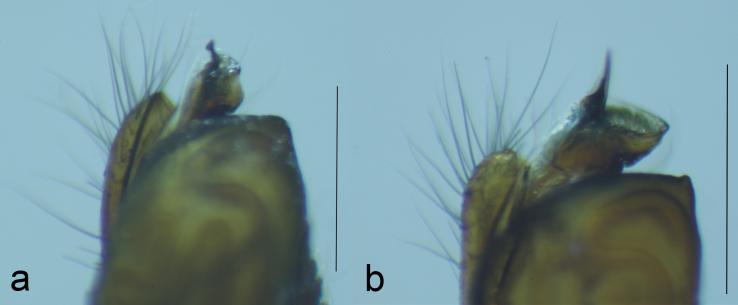
Conductor and embolar tip in (a) *T. ventralis* and (b) *T. sataraensis* (Scale = 0.5 mm).

**Figure 4.**
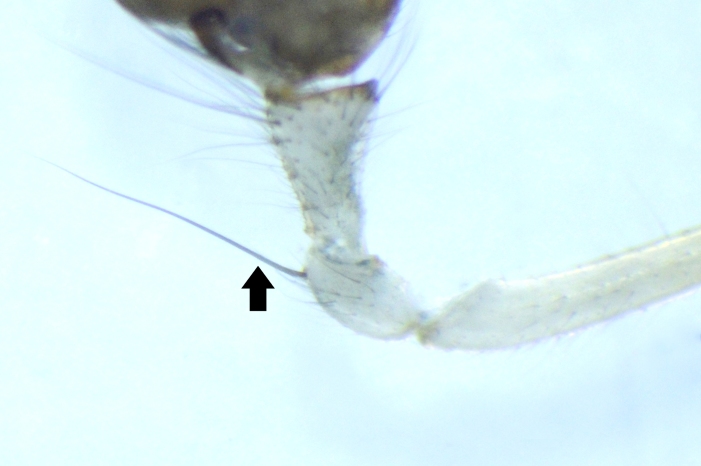
Macroseta on palpal patella in *Tylorida sataraensis*.

**Figure 5.**
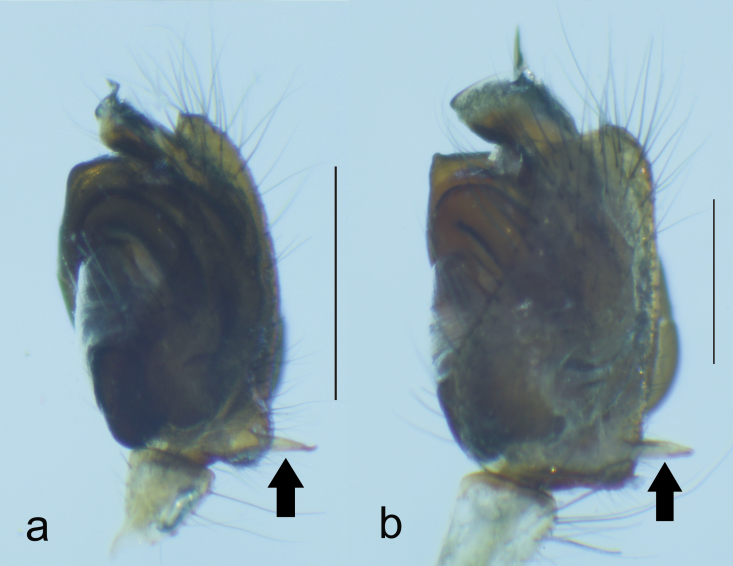
CDBP pointed in (a) *T. ventralis* and (b) *T. sataraensis* (Scale = 1mm).

**Figure 6.**
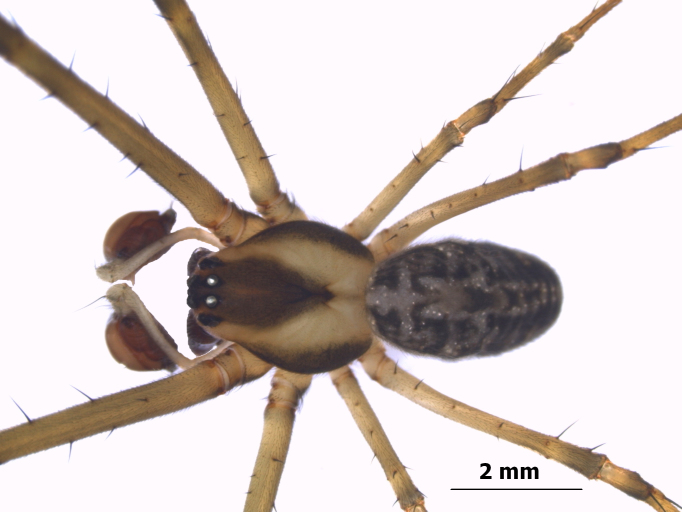
Habitus dorsal view of *Tylorida sataraensis*.

**Figure 7.**
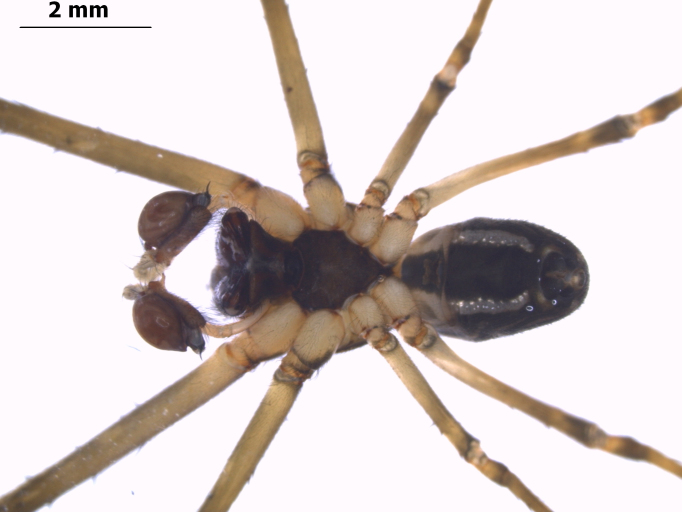
Habitus ventral view of *Tylorida sataraensis*.

*Tylorida sataraensis* is closely related to *T. ventralis* but distinguished from all described *Tylorida* species by the following combination of characters: less swollen tegulum ventrally, longer embolar tip (Figs 1, 2, 3​), presence of macroseta on palpal patella (Fig. 4). CDBP erect and pointed in *T. sataraensis* but slightly bent upwards near tip in *T. ventralis* (Fig. 5). Paracymbium arrow shaped, lateral margins curved, distal end folded inwards in former but with straight lateral margins and distal edge bulged at one end in latter species. Overall, *T. sataraensis* is large sized species than *T. ventralis* (Table 1).

#### Distribution

India, Maharashtra, Satara.

## Discussion

**Figure 8.**
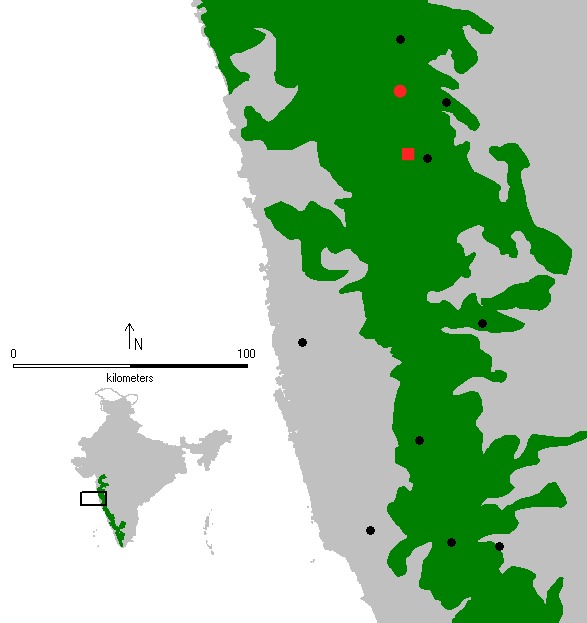
Points showing surveyed rocky outcrop sites in Northern Western Ghats, of which sighting of *T. sataraensis* in red square - Chalkewadi and red circle - Kaas.

**Figure 9.**
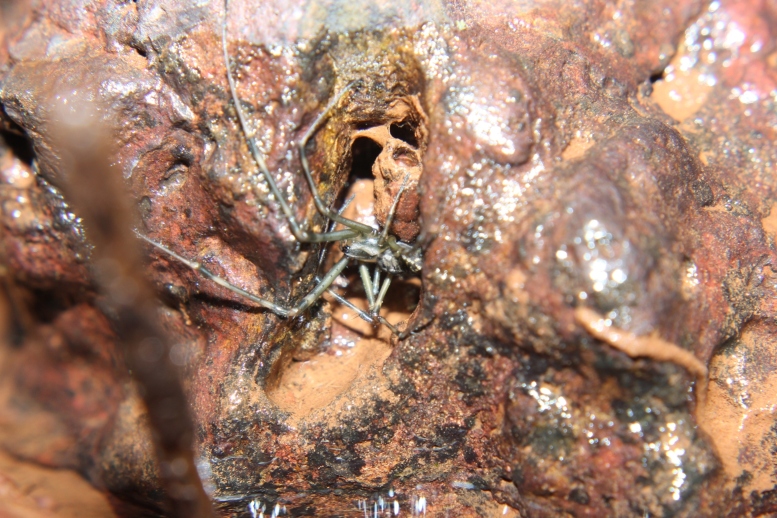
*​Tylorida sataraensis* in a cavity of laterite boulder.

**Figure 10.**
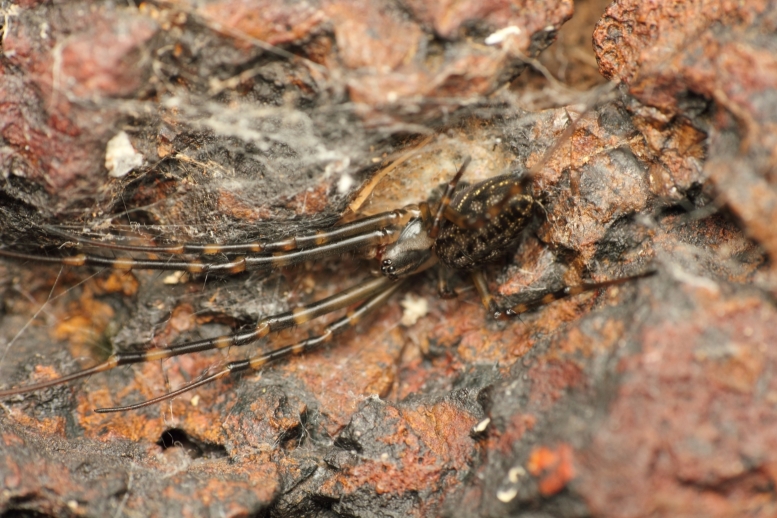
Female *​Tylorida sataraensis* on an egg sac.

**Figure 11.**
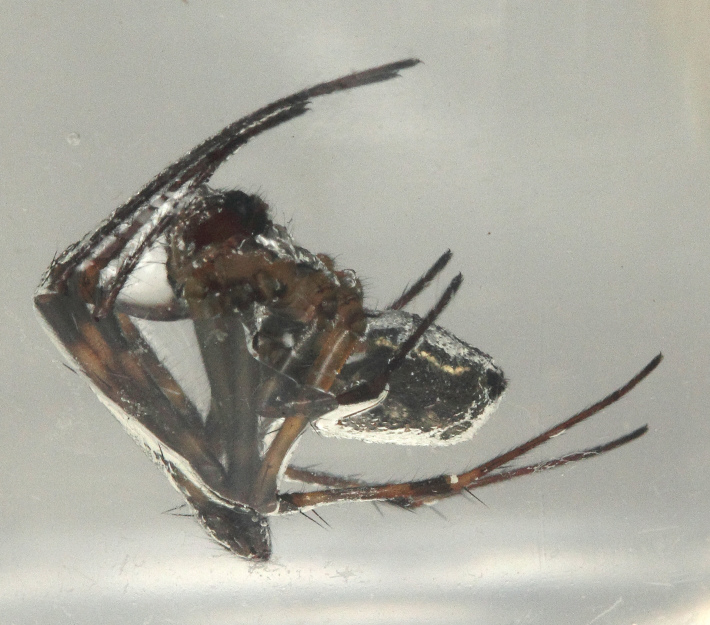
Air trapped on spider body surface.

A total of 309 *T. sataraensis* were observed from three sample surveys across eleven rocky outcrop sites in the northern Western Ghats (Table 2, Fig. 8). *Tylorida sataraensis* was observed only on high altitude plateaus (≈1100 m ASL) inhabiting surface waters of streams shaded by marginal vegetation. All spiders (N=309) were encountered in webs constructed across the channel width of streams. Orb webs were built with silk lines attached to marginal vegetation and laterite rocks. Webs were built high enough (up to 60 cm) and wide enough (up to 100 cm) to avoid fluctuations in stream water levels. Observationally, laterite rocks seemed to be the preferred anchor points for web construction (N=309) and such microhabitat features may influence web site selection for the species. Some specimens (N=56) were also observed hiding on the side of laterite rocks that faced the stream edge during the monsoon. Spiders that were observed on the sides of laterite rocks seemed to prefer using cavities in the rock surface (N=37) for refuge (Fig. 9). Egg sacs (N=12) were laid pre-monsoon, above stream flow, on the side of laterite rocks (Fig. 10). Spiders escaped into the main flow of stream water when disturbed but were anchored by a dragline to adjacent laterite rocks that assisted their return to the web. Air pockets were observed along the body surface of spiders submerged in stream water (Fig. 11) and it is possible such adaptation might assist them to resurface. Twenty spiders were timed underwater displayed significantly different submersion times (t = 5.78, df = 19, P<0.05). An average of 300 seconds was recorded and the most tenacious remained submerged for over 829 seconds (>13 minutes) (Suppl. material 1).

### Conservation status

The greatest current threats to rocky plateau areas continue to be habitat degradation, destruction and direct removal of laterite rocks for construction purposes (Watve 2013). Among those threats observed in the study area, loss of vegetation cover and removal of laterite rocks are likely to be direct threats to *T. sataraensis*. Such microhabitat degradation, if permitted to continue, could reduce the amount of shaded areas and alter the micro-strata required by the species for web construction. Gradual loss of marginal vegetation along streams could also displace spiders and reduce available microhabitat across sites. Where these threats occur, spiders do congregate in patchy pockets where shade is optimal. Whether this is a natural clustering of population or a response to threats/ impacts remains unknown. If such marginalisation of populations are caused by anthropocentric impacts it could potentially fragment populations in the longer term. Local use of stream water for agricultural purposes is also reducing flow levels across some sites. This action could also affect distribution of spiders across each site and alter niche availability.

In total, ten plateaus were surveyed across the breadth of the northern Western Ghats. From these sites, *T. sataraensis* has been observed only at Chalkewadi (type-locality) and Kaas plateaus. Combined these plateaus span an area of only 69 Km^2^. Therefore, in the interests of conservation, we collected only five adult males during the three years survey. Dispersal of these spiders on Chalkewadi and Kaas was observed only during the monsoon period when streams swell and become connected. The extent of occurrence (EOO <100 Km^2^) and area of occupancy (AOO <10 Km^2^) for *T. sataraensis* appears to be fragmented. Based on its current known distribution, the species would likely fall under IUCN status Critically Endangered (CR) following Criteria B1ab(iii)+ B2ab(iii) (IUCN 2012​). The detection of this species so far, at just two localities in the northern Western Ghats, and the observed threats recorded here, suggest that *T. sataraensis* deserves urgent efforts to conserve it.

## Supplementary Material

Supplementary material 1 Submersion time of T. sataraensis under water (N=20) Data type: Time records
File: oo_36566.xlsx Siddharth Kulkarni and Todd Lewis

XML Treatment for Tylorida sataraensis

## Figures and Tables

**Table 1.** Morphometry for diagnosis of male *T. sataraensis* ​(N=6) compared to *T. ventralis* (N=6).

**Character**	**Tylorida ventralis**	**Tylorida sataraensis**
Macroseta on palpal patella	No	Yes
Palpal femora: length to width ratio	9	10-10.5
Tibia length to width ratio	1.15	1.5
Paracymbium to CDBP ratio (range)	0.60-0.75	0.95-1
Embolus: CDBP ratio	0.5	0.97-1
Total size (range in mm)	6.17-7.58	8.1-9.7 (larger species)
Leg I length to carapace length ratio (range)	10.2- 11.71	6.9-7.6

**Table 2.** Abundance from three sample surveys of *T. sataraensis*.

**Survey**	**Males**	**Females**	**Juveniles**	∑
Jan-14	8	31	64	103
Jun-14	10	18	56	84
Sep-14	39	64	19	122
∑	57	113	139	309
